# P-531. Overlap of Respiratory Virus Detection from Concomitant Air and Human Specimens in Elementary School Classrooms in Kansas City, Missouri

**DOI:** 10.1093/ofid/ofaf695.746

**Published:** 2026-01-11

**Authors:** Jennifer E Schuster, Brian R Lee, Nibha Sagar, Brittney Fritschmann, Anjana Sasidharan, Luke C Gard, Dithi Banerjee, Olivia Almendares, Hannah L Kirking, Rangaraj Selvarangan, Jennifer Goldman

**Affiliations:** Children's Mercy Kansas City, Kansas City, MO; Children's Mercy Kansas City, Kansas City, MO; Children's Mercy hospital, Kansas City, Missouri; Children's Mercy Hospital, Kansas City, Missouri; Childrens Mercy Hospital, Missouri, Kansas; Children's Mercy Hospital-Kansas City, Kansas City, Missouri; Children's Mercy Hospital, Kansas City, Missouri; Centers for Disease Control and Prevention, Atlanta, Georgia; Coronavirus and Other Respiratory Viruses Division, National Center for Immunization and Respiratory Diseases, CDC, Atlanta, GA; Children’s Mercy Hospital, Kansas City, Missouri; Children's Mercy Hospital, Kansas City, Missouri

## Abstract

**Background:**

Little data are available describing the circulation of respiratory viruses in pre-kindergarten (preK)-12^th^ grade schools. Performing viral surveillance testing in students and staff can be resource intensive and requires large scale participation. Air sampling for respiratory viruses in schools could be a proxy to human sampling. Our objective was to describe the overlap of respiratory viral detection in human and air specimens collected concurrently.

**Figure:**
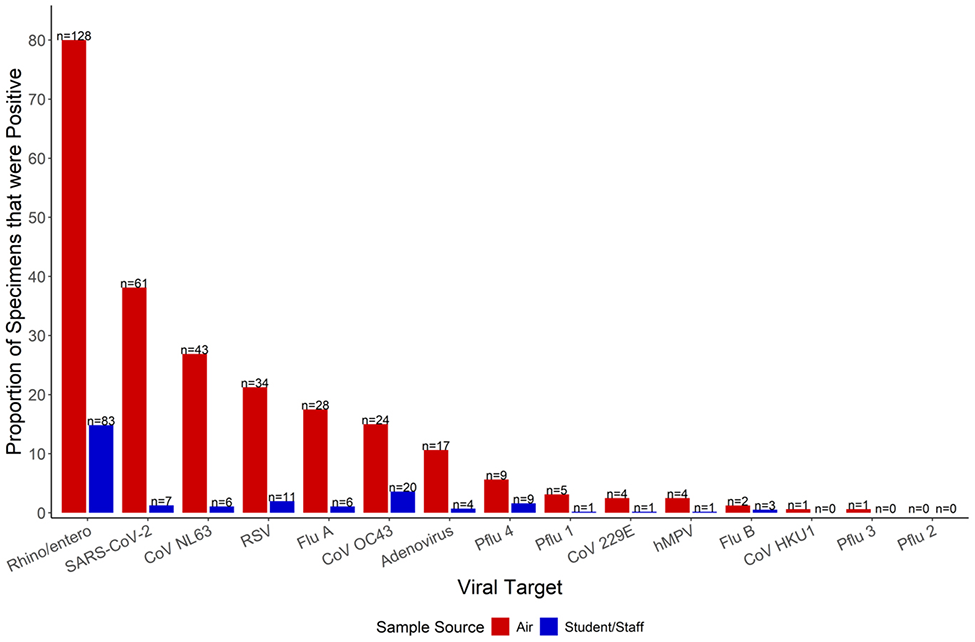
Respiratory viruses detected from air samples and nasal swab specimens in elementary schools in Kansas City, MO from September 2024- March 2025.

**Figure 2.**
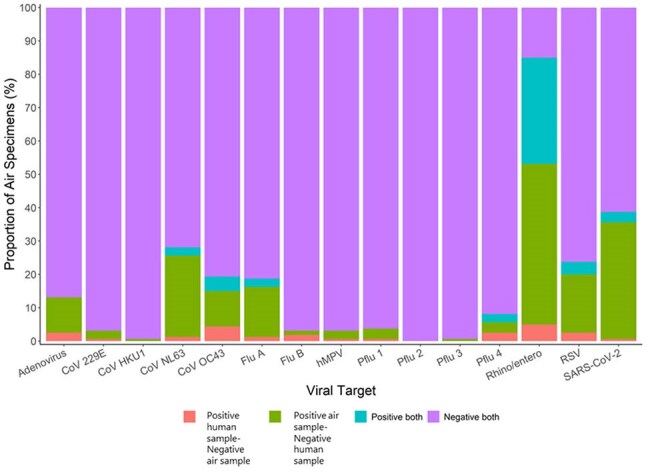
Concordance of respiratory virus detections from matched air and human specimens in elementary schools in Kansas City, MO from September 2024-March 2025.

**Methods:**

School KIDS is a prospective respiratory virus surveillance program for students and staff in a preK-12^th^ grade public school district in Kansas City, MO. In 15 classrooms across 5 elementary schools (3 classrooms/school), human (anterior nares) specimens were collected monthly; air samples were collected on the same day every 2-4 weeks using AerosolSense samplers (ThermoFisher Scientific) operating at 200 L/minute for 8 school hours. All specimens were tested by PCR for adenovirus, human metapneumovirus, influenza, parainfluenza viruses 1-4, respiratory syncytial virus, rhinovirus/ enterovirus (RV/EV), seasonal coronaviruses (sCoV) OC43, NL63, HKU1, 229E, and SARS-CoV-2. Concordance—defined as detection of the same virus in both air and human samples from the same room and day—was assessed.

**Results:**

From September 18, 2024-March 31, 2025, 160 air samples were collected concurrently with 561 human specimens, with a median of 3 [IQR 3,5] human specimens per air sample. Viruses were detected in 142/160 (89%) air samples, with a total of 348 viral detections. Of 160 air samples, 64 (40%) had the concurrent detection in at least one match human specimen. Of 561 human samples, 104 (18%) had the same virus as in matched air samples. Of the 561 human specimens, 144 (25%) had ≥1 virus detected with a total of 152 viral detections. RV/EV (128/160) and SARS-CoV-2 (61/160) were the most frequently detected viruses in air, while RV/EV (83/561) and sCoV OC43 (20/561) were the most frequently detected in human specimens (Fig 1). RV/EV (n=51) and sCoV OC43 (n=7) were the viruses most frequently identified from matched human and air samples (Fig 2).

**Conclusion:**

Air sampling in schools may be useful to detect circulating viruses in schools. Further studies are needed to determine whether air sampling can be a proxy for human sampling.

**Disclosures:**

Brian R. Lee, PhD, MPH, Merck: Grant/Research Support Rangaraj Selvarangan, PhD, Altona: Grant/Research Support|Biomerieux: Advisor/Consultant|Biomerieux: Grant/Research Support|Biomerieux: Honoraria|Cepheid: Grant/Research Support|Hologic: Grant/Research Support|Hologic: Honoraria|Meridian: Grant/Research Support|Qiagen: Grant/Research Support

